# Selective Trace Mix: A New Processing Tool to Enhance Seismic Imaging of Complex Subsurface Structures

**DOI:** 10.3390/jimaging12030124

**Published:** 2026-03-12

**Authors:** Mohamed Rashed, Nassir Al-Amri, Riyadh Halawani, Ali Atef, Hussein Harbi

**Affiliations:** 1 Water Research Center, King Abdulaziz University, Jeddah 80200, Saudi Arabia; 2 Geology Department, Suez Canal University, Ismailia 41522, Egypt; 3 Faculty of Environmental Sciences, King Abdulaziz University, Jeddah 80200, Saudi Arabia; 4 Department of Geophysics, Faculty of Earth Sciences, King Abdulaziz University, Jeddah 80200, Saudi Arabia

**Keywords:** processing, coherence, imaging, fault, fold

## Abstract

In seismic imaging, the trace mixing process involves merging neighboring traces in seismic data to enhance the signal-to-noise ratio and improve the continuity and spatial coherence of seismic data. In regions with complex subsurface structures, current trace mix filters are often ineffective as they introduce artifacts that reduce interpretability and obscure the signatures of important structures, such as faults and folds. We introduce the selective trace mix as a novel, data-dependent filter. This filter enhances amplitude consistency, spatial coherence, and the definition of reflections, while it preserves complex structures and maintains their clarity. Selective trace mix uses sequential steps of evaluation, referencing, exclusion, weighting, and normalization of all samples within the filter operator. As a result, selective trace mix is a temporally and spatially variable, data-dependent filter. The filter’s effectiveness is validated using both synthetic and real field seismic data. Synthetic data is a portion of the Marmousi seismic model, while real data include land and marine seismic datasets imaging complex subsurface fault/fold structures. When compared to three of the commonly used conventional filters, the selective trace mix yields far better results in terms of horizon integrity and fault clarity.

## 1. Introduction

Trace mixing is a well-established seismic processing filter that combines and manipulates individual seismic traces to enhance data quality. The basic idea of trace mixing is to merge a group of neighboring traces into a new trace and replace the middle trace with the new one. The commonly used operator width of trace mixing usually ranges between 3 and 11 traces [1]. This new trace carries characteristics of its neighbors, which improves spatial coherency and lateral continuity of reflections. Various trace mixing techniques help mitigate issues like noise, irregularities, and deficiencies in seismic data [2].

The most basic and simplest form of trace mixing is the straight mean trace mix (SMTM). In SMTM, the arithmetic mean of a selected group of traces within a user-defined window is calculated and assigned as the middle trace in that window. The window slides horizontally, and the process is repeated till the end of the seismic section. This simple process can be expressed by Equation (1):

(1) $$A\left(t\right)=\frac{1}{N}\sum_{i=1}^{N}a_{i}(t)$$

where *A*(*t*) is the sample value of mixed trace at time *t*, *N* is the number of traces within the operator, and $a_{i}$ is the sample value of the *i*th trace at time *t* [1].

The SMTM filter yields satisfactory results for seismic data with horizontal reflections; however, it fails to produce accurate results on sections with reflection offsets caused by faulting or curvature from folding. Noise bursts can significantly damage trace mixing. This is mainly because SMTM lacks a discrimination function and assigns equal weights within the operator window. To address this, the weighted mean trace mix (WMTM) is introduced. WMTM assigns a specific weight to each sample within the operator, based on various criteria, before calculating the arithmetic mean of the weighted samples [3,4]. This process is shown by Equation (2):

(2) $$A\left(t\right)=\frac{\sum_{i=1}^{N}W_{i}a_{i}}{\sum_{i=1}^{N}W_{i}}(t)$$

where $W_{i}$ is the assigned weight for the sample value $a_{i}$ of the *i*th trace at time *t*.

This allows the use of different weighting functions, such as assigning a weight of one to the middle sample and ramping to zero at the edges, Gaussian ramping to the edges’ samples, or manually assigning weights to individual samples within the operator window. This gives the processor considerable maneuvering capability and the ability to manipulate the trace mixing process to suit the seismic data being processed. However, in both the SMTM and WMTM filters, anomalously high or low amplitudes have a damaging effect on the trace mixing process, and the results are not always satisfactory.

The median trace mixing (MDTM) filter utilizes the median instead of the arithmetic mean or the weighted mean to mitigate the issue of noise bursts. In MDTM, samples within the operator window are arranged in ascending or descending order, and the sample in the middle is assigned as the representative sample in the mixed trace [5]. This is performed using Equation (3):

(3) $$A\left(t\right)=median(a_{1t},a_{2t}\;,\ldots \ldots \ldots ,\;a_{Nt})$$

where *A*(*t*) is the sample value of mixed trace at time *t*, *N* is the odd number of samples within the operator window, and $\left(a_{1t},a_{2t}\;,\ldots \ldots \ldots ,\;a_{Nt}\right)$ are the samples value at time *t* sorted in ascending or descending order [6].

Although MDTM addresses the issue of anomalously high or low amplitudes that deviate from the rest of the amplitudes, it has its drawbacks. One of the major drawbacks of this filter is the abrupt changes in amplitude between consecutive samples in the mixed trace, which cause significant waveform distortion in the seismic section after trace mixing. To overcome the distortion problem arising from selecting the single median value in the MDTM, the alpha-trimmed trace mix (ATTM) filter calculates the arithmetic mean of several values around the median. In the ATTM filter, the sample value is calculated using Equation (4):

(4) $$A\left(t\right)=\frac{1}{N-2L}\sum_{i=L+1}^{N-L}a_{i}(t)$$

where *N* is the number of ordered samples within the trace mix operator, $a_{i}$ is the amplitude of the *i*th sample, α is the trimming parameter (0 < α < 0.5) and *L* = [*α N*]. When α equals zero, all samples within the operator window are included in the mixing process and *A*(*t*) is equal to the conventional straight mean trace mix. When α equals 0.5, only the middle value within the operator window is considered and *A*(*t*) is equal to the median stack. By adjusting the parameter α, a trace is obtained that exhibits properties of both the MDTM filter and the SMTM filter [7,8].

All the previously discussed filters share two common disadvantages: filter stationarity and processor influence. Filter stationarity is inherent, given the fixed design and constant parameters of the trace mixing filter applied to the entire seismic section under processing. While the filter may be suitable for a portion of the seismic data, it is unlikely to be suitable for the entire dataset. This is attributed to the variable nature of seismic data. Seismic data are subject to temporal and lateral changes in waveform, amplitude, frequency, and other attributes. These changes are attributed to the heterogeneous and anisotropic nature of subsurface rocks [9]. Changes in the characteristics of seismic data may arise from various factors, including but not limited to changes in the lithology and composition of subsurface rocks, their fluid contents, porosity, permeability, thickness, and geologic structures such as faults, folds, and unconformities [10,11].

The processor interference issue arises from the fact that selecting the trace mixing filter parameters is based solely on the processor’s experience and personal bias. The percentage or number of excluded samples and the weights assigned to the remaining ones are selected by the processor, which is both a tedious and time-consuming task. Both filter stationarity and processor interference problems hinder the application of trace mixing filters to post-stack seismic data, particularly for those with complex subsurface structures. Conventional trace mixing filters may reduce the spatial resolution of seismic data, especially where lateral structural or stratigraphic variations are large [12]. These trace mixing filters can have a destructive effect on seismic data and may introduce artifacts that may lead to misinterpretation. This is especially true in areas with complex subsurface structures, where seismic sections have steeply dipping layers, faults, and/or folds. The proposed selective trace mixing (STM) filter addresses these issues, as the filter parameters are dependent on the nature of the seismic data being processed and change as the trace mix operator slides both vertically and horizontally.

## 2. Materials and Methods

The process of selective trace mixing (STM) is a sequence of data evaluation, referencing, exclusion, weighting, and normalization of samples’ amplitudes within the mixing operator window at every operator step. This sequential process aims to guarantee that only samples of similar characteristics are mixed, while samples of different characteristics, especially amplitude, are either excluded from the mix or downweighed in the mixing process. The STM also allows the parameters to vary as the filter’s operator slides temporally and spatially according to the nature of the samples within the operator, and hence the variability of the filter. The following is a description of the STM procedures.

- For every number of samples within the user-defined trace mix window, define the reference sample, which is the sample that best represents the most amplitudes within the window. Both the median and alpha-trimmed mean, with high percentage exclusion, can be used for this purpose. Our experiments have demonstrated that both methods typically yield satisfactory results.
- Exclude all samples having different signs from the reference sample, as shown in the following equation:

(5) $$\hat{a_{i}}\left(t\right)=\left\{\begin{array}{l} a_{i}\left(t\right)\;\;if\;\;\;sign\;\left(a_{i}\left(t\right)\right)=sign\;\left(a_{r}\right)\;\;\;\;\;\;\;\;\;\;\;\;\;\;\;\;\;\;\;\;\;\;\\ \;\;\;\;\;\;\;\;\;\;\;\;\;\;\;\;\;\;\;\;\;\;\;\;\;\;\;\;\;\;\;\;\;\;\;\;\;\;\;\;\;\;\;\;\;\;\;\;\;\;\;\;\;\;\;\;\;\;\;\;\;\;\;\;\;\;\;,1\leq i\leq N\\ Null\;\;\;\;\;\;\;\;\;\;\;\;\;\;\;\;\;\;\;Otherwise\;\;\;\;\;\;\;\;\;\;\;\;\;\;\;\;\;\;\;\;\;\;\;\;\;\;\;\;\;\;\;\;\;\;\; \end{array}\right.$$

where $\hat{a_{i}}\left(t\right)$ is the selected samples within the operator window whose number is *M*, and *M* ≤ *N*.

When samples with opposite amplitudes are mixed, they tend to cancel each other and cause amplitude reduction in the mixed sample. Therefore, this step aims to avoid including samples with opposite amplitudes to the reference sample from the mixing process. These samples occur when reflections have drastic vertical shifts, causing samples of opposite amplitudes to align. An example of this occurs as the operator slides across the cross-over point of fault planes or near the tip of a fold.

- Calculate a rank for every remaining sample within the operator window based on its closeness to the reference sample, using Equation (6):

(6) $$X_{i}=\sqrt{{\left(a_{i}\left(t\right)-a_{r}\right)}^{2}}$$

where $X_{i}$ is the rank of sample $a_{i}\left(t\right)$ that represents the closeness of the sample to the reference sample. The smaller $X_{i}$ the closer the sample to the reference sample.

- With the aid of the ranks calculated in the previous step, assign a specific weight ($W_{i}(t)$) for each of the remaining samples within the operator window, using the following equation:

(7) Wi(t)=1Xi2

Some samples may have similar sign of the reference sample but have anomalously high or low amplitudes. Accordingly, this step is designed to ensure that samples whose amplitude signs match those of the reference sample, but whose values are anomalously far from it are given lower weights and hence contribute less to the mixed trace. Because there is a small possibility that *Xi* = 0, the algorithm checks for such a rare condition, and adds a tiny value to the *Xi* in such a case.

- Normalize the calculated weights so that the summation of weights equals 1 by dividing each calculated weight by the sum of weights, using the following equation:

(8) $${\hat{W}}_{i}(t)=\frac{W_{i\;(t)}}{\sum_{i=1}^{M}W_{i}(t)}$$

where ${\hat{W}}_{i}(t)$ is the normalized wight of the *i*th sample at time *t*, and *M* is the number of samples after exclusion within the operator window. The weight normalization process aims to preserve the original amplitudes of seismic data.

- Multiply the normalized weights by the corresponding sample amplitudes, using the following equation:

(9) $$\hat{a_{i}^{w}}\left(t\right)={\hat{W}}_{i}\left(t\right).{\hat{a}}_{i}(t)$$

By the end of this step, samples that may harm the mixing process are excluded, and samples with anomalously high or low amplitudes are downweighed, ensuring that only useful samples are the primary contributors to the new mixed trace.

- Finally, calculate the arithmetic mean of the remaining normalized weighted samples within the operator window, using Equation (10), and assign this value to the central value within the operator window:

(10) $$A\left(t\right)=\sum_{i=1}^{M}\hat{a_{i}^{w}}\left(t\right)$$

- Slide the operator to the next position, both vertically and horizontally until the entire seismic section is processed. The resultant section is a selective trace mixed section.

The previously discussed procedures of the selective trace mixing algorithm can be summarized in four major steps. The first step is to evaluate samples within the operator window and to assign a reference sample that represents the central portion of the samples within that window. The aim is to find an amplitude value that represents the majority of amplitudes in the window and is least influenced by marginal samples, which are usually a result of vertical shifts in reflections caused by complex structures. The second step is to exclude samples with different signs from the mixing process, as these samples are most likely to occur when the operator approaches a complex geologic feature, such as the crossover point of a fault, folded layers, or steeply dipping layers. The third step involves grading, weighting, normalizing, and averaging the remaining samples within the window to obtain a specific normalized weight for each sample within the operator window. The fourth and last step is to multiply each remaining sample by its assigned normalized weight. This way, the original amplitudes are preserved, and the harmful smearing effect in areas with sudden variations, such as complex structures, is avoided.

**Figure 1:**
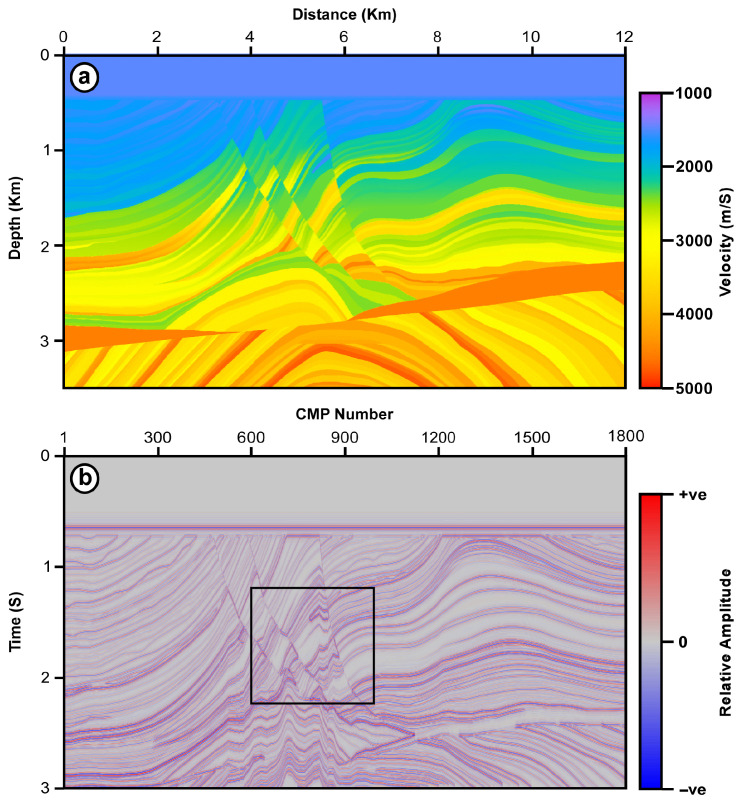
Marmousi2 model (**a**) and its synthetic seismic response (**b**). The black rectangle marks the portion of the data used for testing the proposed STM filter.

The proposed selective trace mix (STM) filter is tested by comparing its results with those of the SMTM, MDTM, and ATTM filters. These four filters are applied to a synthetic dataset and two different seismic sections. The synthetic dataset is generated using the Marmousi2 model (Figure 1a).

The Marmousi2 model [13] is an updated version of the original Marmousi model [14]. The original Marmousi model is a complex 2D structural model with strong horizontal and vertical velocity variations. On the other hand, the Marmousi2 model is an expansion of the original model in both directions. It is also a transformation of the original model into a fully elastic model, with high-frequency, high-fidelity, elastic, finite-difference synthetics (Figure 1). A relatively small portion of the seismic data, between time intervals 1200–2200 ms and CMP numbers 600–1000, is selected to examine the performance of the proposed STM filter and compare it to the other three filters. This portion of the data is chosen because it contains complex structures including faulting, folding, and layers dipping with different dip angles (Figure 1b).

**Figure 2:**
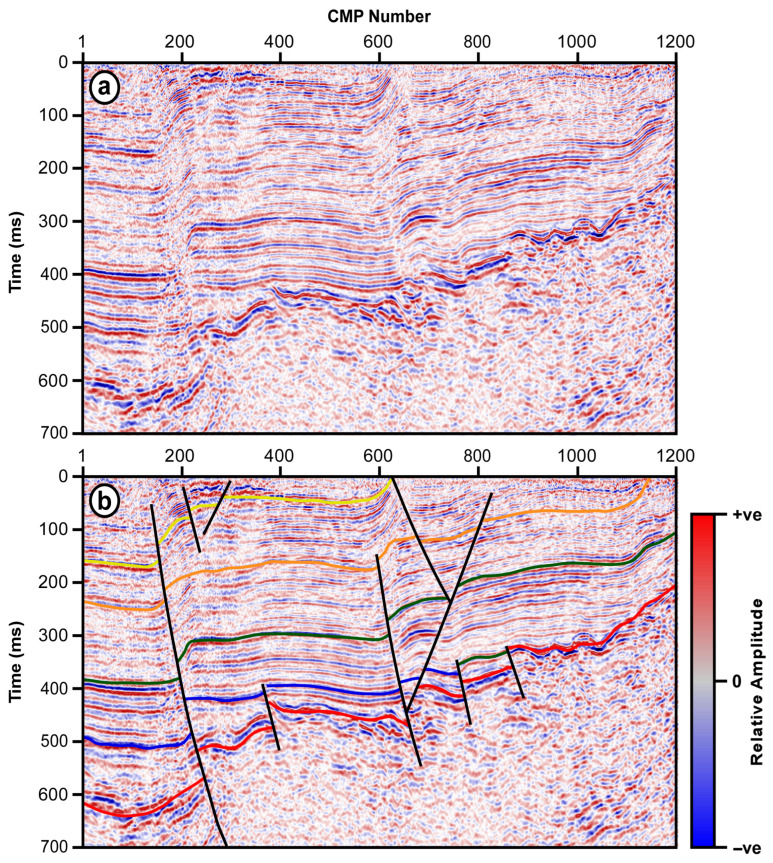
Nara land seismic section (**a**) and its interpretation (**b**). The interpreted section shows the faulted brittle basement rocks, overlain by ductile sedimentary succession of alternating marine and non-marine sediments, and the reverse faults affecting them.

The first field seismic section is part of a land seismic survey conducted over a faulted area in Nara Basin, Japan (Figure 2a). The section is collected over an area with alternating marine and non-marine clay and sand layers overlying complex granitic basement rocks. Both the ductile sedimentary clay and sand layers and the brittle granitic complex underneath are repeatedly thrusted, creating a complex subsurface structural situation (Figure 2b).

The Nara land seismic line is acquired using a JIM-200 P-wave impactor (JIMTech, Tehran, Iran). The data are collected using six in-line geophone arrays spaced 10 m apart, with a shot interval of 5 m, using a 60-channel Bison-9060A system (Bison Instruments, Inc., Lake Park, MN, USA) with a sampling interval of 1.0 milliseconds. A wide-band-pass filter is applied during data recording. This seismic line is subjected to a simple processing routine of field geometry, routine editing, static correction, automatic gain control, predictive deconvolution, and frequency filtering. The data are then subjected to velocity analysis, normal move-out (NMO) correction, and stacking.

**Figure 3:**
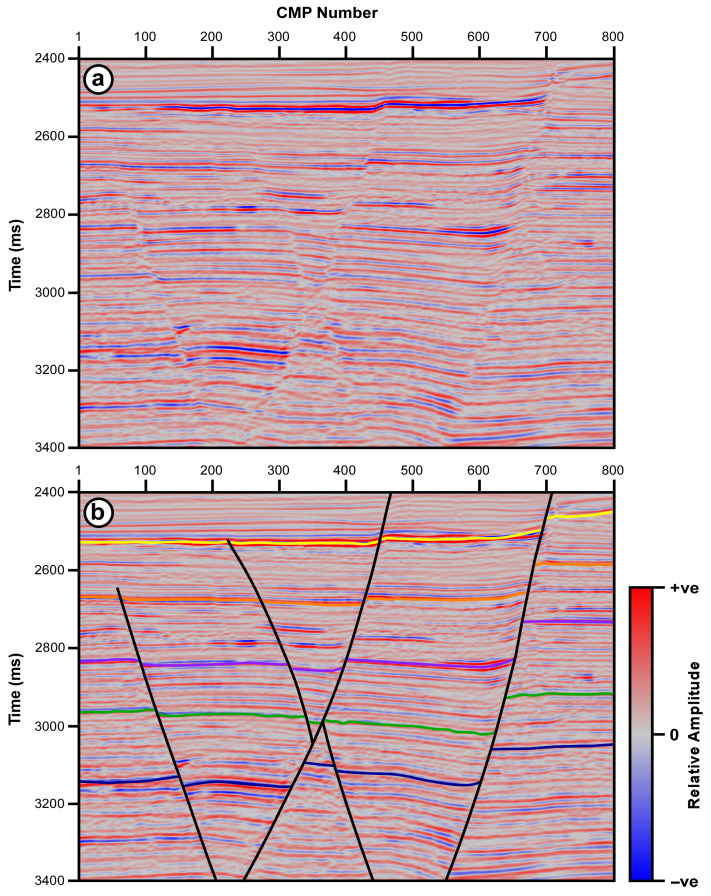
DUG Insight marine seismic section (**a**) and its interpretation (**b**). The interpreted section shows normal faults dipping in opposite directions forming horst structure.

The second seismic section is part of a sample seismic dataset provided by DUG Insight seismic data processing, interpretation, and visualization software [15]. The selected section is a part of marine seismic data collected over a highly faulted area (Figure 3a). The section displays a group of diagonal intersecting faults that dip in opposite directions, forming a graben-like structure (Figure 3b).

## 3. Results

This section presents the results of applying the proposed STM filter and the commonly used SMTM, MDTM, and ATTM filters to the synthetic data and both the Nara land seismic section and the DUG Insight marine seismic sections.

### 3.1. Synthetic Data

**Figure 4:**
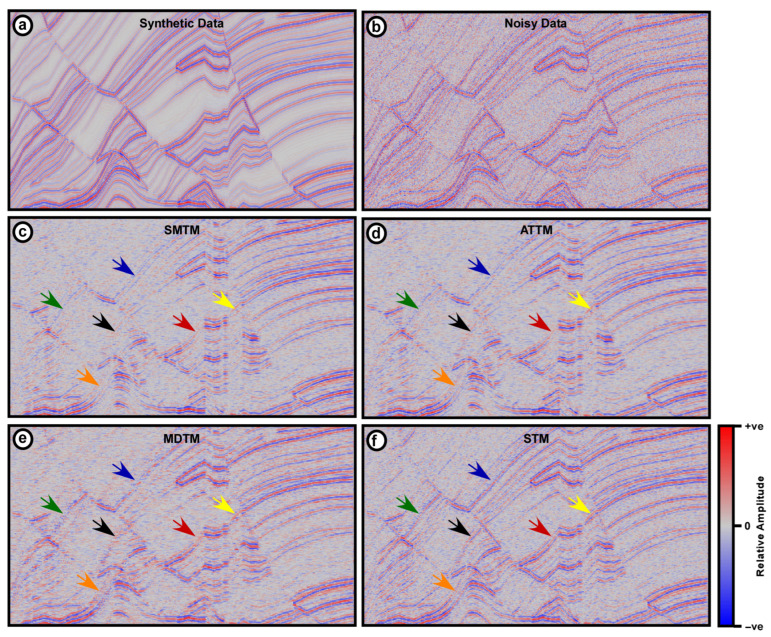
Selected Marmousi2 synthetic seismic data (**a**), the same data after adding Gaussian noise (**b**), and the outputs of applying different trace mixing filters (**c**) SMTM, (**d**) MDTM, (**e**) ATTM, and (**f**) STM. Colored arrows point to areas of significant differences between STM and other filters.

The selected portion of the Marmousi2 synthetic seismic data images a complex subsurface structure comprising three semi-parallel normal faults with folded layers dipping in different directions with different dip angles (Figure 4a). To mimic real field data with horizontal and vertical amplitude variations, 10% Gaussian noise is added to the test synthetic data (Figure 4b). Although not perfect representation of real field seismic data, this is best available solution when using perfectly simulated data. The operator width used for the four filters is 7 traces and the trimming alpha value for both the ATTM and STM filter is 0.27. Figure 4a shows the synthetic data portion chosen for testing and Figure 4b shows the same data after adding Gaussian noise. The results of applying the SMTM, ATTM, MDTM, and the proposed STM filters are displayed in Figure 4c–f.

The three conventional filtering tools provide significantly different outputs from the output of the proposed STM filter. In terms of the overall clarity, the STM output provides high clarity compared to other filters. A good example of this is the dipping reflection indicated by the blue arrow. This reflection appears distorted and disrupted on the MDTM output and can be hardly seen on the SMTM and ATTM outputs. On the contrary, the reflection appears intact, continues and can be easily traced in the STM output. The same scenario is true in most weak dipping reflections, such as those indicated by the black and green arrows.

The discrimination against random Gaussian noise is almost similar in the outputs of the four filters with a slight advantage of the proposed STM filter. MDTM filter yielded distorted section infested with sudden amplitude variations in both the vertical and horizontal directions. However, since suppressing random noise is not the main aim of trace mixing filters, this could be considered an advantageous byproduct of the STM filter.

Reflection continuity is the real judgement of the performance of trace mixing filters, as it is the main objective of the mixing process. Figure 4 clearly shows large difference between the high reflection continuity of the STM section and other sections. This is indicated by the reflections marked by the orange arrow pointing to reflections on the limb of the fold. Sharpness is another aspect of judging the filtering outputs. Reflections marked with the yellow, blue, and green arrows are good examples of reflections that appear sharp on the STM output, while fuzzy and faded on the other filtering outputs.

Fault definition on the STM output is much higher compared to other filtering outputs. Reflection terminations are more distinguishable clearer than in other filtering outputs. This is best seen in the area marked by the red arrow (Figure 4).

A great benefit of testing a new tool and comparing its performance against other tools using synthetic data is the capability to compare the different outputs to the noise-free data. This allows acquiring solid quantitative parameters such as Root Mean Square Error (RMSE) and correlation coefficients between the reference data and different outputs. RMSE and correlation coefficients of the four filtering outputs are listed in Table 1. The STM exhibits the lowest RMSE of 0.9750, followed by the MDTM with an RMSE of 1.0389. The RMSE of the SMTM and ATTM are significantly higher, with an RMSE of 1.7302 and 1.6548, respectively. Moreover, STM shows the highest correlation coefficient with the reference data of 0.8178. On the other hand, SMTM, ATTM, and MDTM have correlation coefficients of 0.7161, 0.7185, and 0.6927, respectively (Table 1). Although synthetic data do not resemble the lateral and temporal amplitude variability, they could provide a preliminary quantitative evaluation of the proposed STM filter compared to other filters.

### 3.2. Land Data

**Figure 5:**
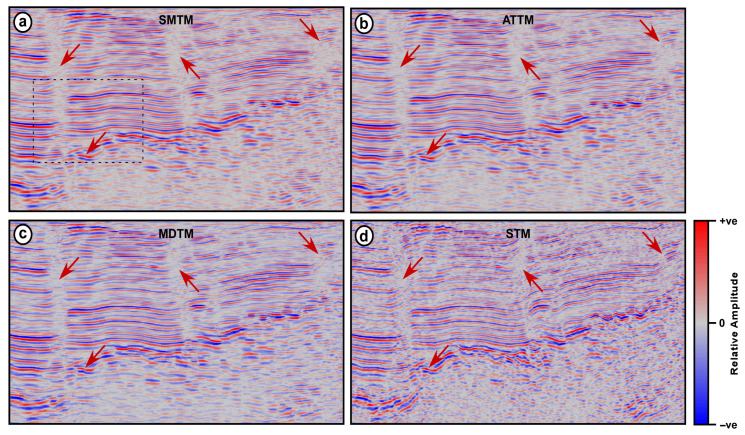
Nara land seismic section after applying different trace mixing filters (**a**) SMTM, (**b**) MDTM, (**c**) ATTM, and (**d**) STM. The red arrows point to areas of significant differences, and the dotted black rectangle indicates the area magnified in Figure 4.

The Nara seismic section is collected over an area with a complex subsurface geology, with two reverse faults cutting through basement rocks overlayed by a sequence of marine and nonmarine sediments. The four above-mentioned trace mixing filters are applied to the seismic section for comparison (Figure 5). An operator width of 9 traces is used for the four filters, while an alpha value of 0.3 is used for both the ATTM filter and for calculating the reference value in the STM filter.

Both the SMTM and ATTM filters resulted in degraded seismic sections with over-smoothed reflections, and many geological features were lost (Figure 5a,b). For example, in these two sections, the thrust fault near the left edge of the section is blurred, apparently due to the mixing of samples with opposite amplitudes within the fault zone. Another example of distorted geologic features is the basement horizon. In these two sections, the basement is ill-defined, lacks lateral coherency, and is highly distorted (Figure 5a,b).

The MDTM filter, however, yields a slightly improved seismic section, where geological features are less distorted (Figure 5c). The induced high-frequency noise and the abrupt amplitude changes in almost all reflections reduce the spatial coherency of seismic signals and complicate the interpretability of the seismic section. The inconsistent amplitudes and the rough reflections resulting from the MDTM filter are visible in the magnified region shown in Figure 5c.

On the contrary, the seismic section resulting from the STM filtering maintains clear, coherent, and consistent reflections over the entire section (Figure 5d). In the output of the STM filter, the basement horizon appears as a strong and continuous reflection, unlike the outputs of the other three filters. Moreover, the reflections of the overlying sedimentary layers are strong, distinct, and coherent. Both thrust faults near the two ends of the section are sharp, easily traceable, and show clear vertical displacements (Figure 5d).

The red arrows in the upper left corner of the seismic sections point to a thrust fault (Figure 5b). In the sections resulting from the SMTM, MDTM, and ATTM filters, a notable amplitude loss is observed. On the other hand, the section resulting from the STM filter maintains the fault integrity, where the sag and drag effect of the fault on the ductile sediments is clearly visible. The dipping layers in the upper right corner of the seismic section, and the basement-sediments interface in the lower left corner of the sections are better preserved in the STM section. Moreover, a careful examination of the fault in the middle of the seismic section reveals that the STM is more effective in preserving fault integrity, as evidenced by reflection termination and reflection characteristics on both sides of the fault (Figure 5d).

**Figure 6:**
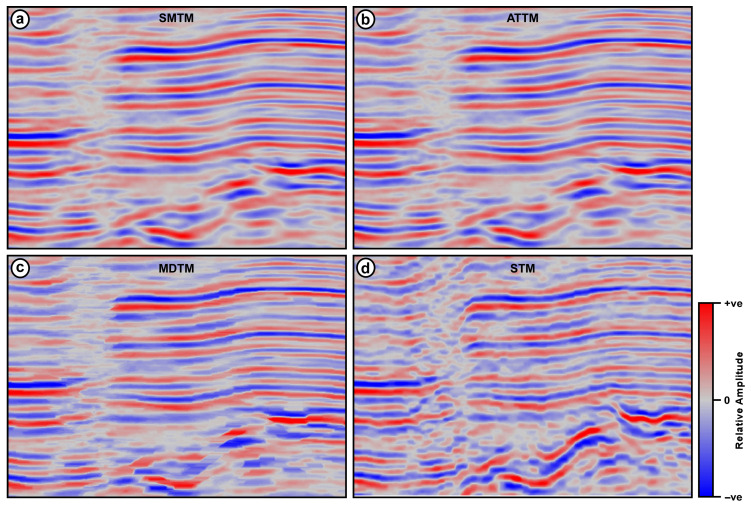
Magnified views of the area marked with the dotted black rectangle in Figure 5. (**a**) SMTM and (**b**) ATTM show smearing and interfering horizons, (**c**) MDTM shows abrupt amplitude changes, while (**d**) STM shows clear noise reduction and horizon integrity preservation.

The magnified views of portions of the four seismic sections resulting from the four filtering techniques are shown in Figure 6. These magnified views confirm that the output of the STM filter has clear, interpretable data with well-defined and coherent reflections on both sides of the fault (Figure 6d). This is not the case in the other 3 outputs of the conventional filters (Figure 6a–c).

**Figure 7:**
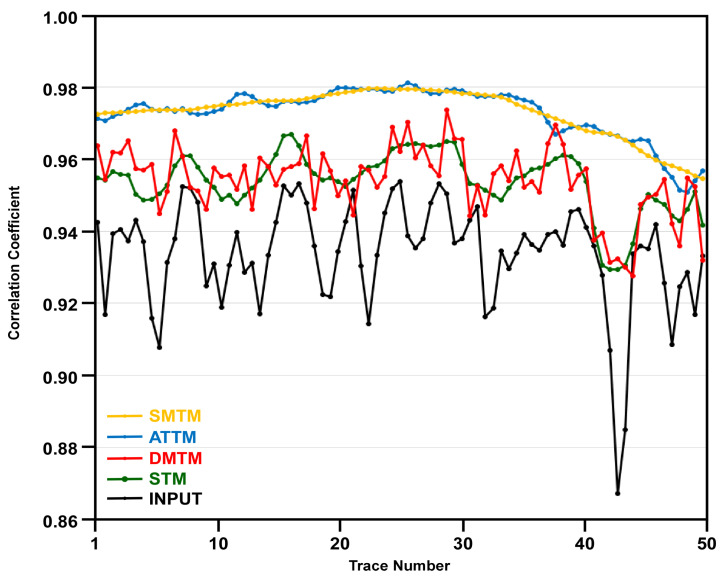
Plot of correlation coefficients between adjacent traces within a selected region with almost horizontal reflections in the input data and the outputs of the four filters for the Nara section.

Visual inspection and interpretability of a seismic section are the cornerstone of evaluating the performance of a new filter or a processing tool. However, quantitative analysis of the filtering output is also of great importance. The correlation coefficient between adjacent traces is a measure of the lateral coherency of reflections in seismic data [14,15,16,17,18]. This rough estimation of lateral coherency in seismic sections is especially valid when calculated for areas with horizontal reflections [19]. Accordingly, a small area with mostly horizontal reflections is selected from the input data and the four filtering outputs. This area lies between the time intervals 300 to 420 ms and the CMP numbers 411 to 460. The correlation coefficient is calculated between each 2 adjacent traces in the 5 datasets, and the results are plotted in Figure 7.

Both the SMTM (yellow) and ATTM (blue) filters have higher correlation coefficients, but they have no correlation to the input data. These extremely high correlation coefficients indicate that both SMTM and ATTM filters have resulted in traces where the mixing process is so aggressive that it has led to great similarity among traces, at the expense of the individual characteristics of original traces. This is indicated by the smooth yellow and blue curves, which have no correlation with the input data curve in black (Figure 7).

On the other hand, the output of the MDTM (red) filter still maintains the characteristics of the input data; however, it exhibits drastic changes from one point to the next. This is caused by the inherent issue of the median filter’s reliance on a single value. On the contrary, the STM (green) filter has resulted in traces with higher correlation coefficients than the input data, while maintaining a similar trend, indicating the effectiveness of the STM filter in enhancing the spatial coherence of reflection while preserving the original characteristics of the input data (Figure 7).

Root Mean Square Error (RMSE) can be used to measure the similarity between the input data and each of the four filtering outputs. Accordingly, RMSE is calculated to quantify the error the average magnitude of errors between the correlation coefficients of the input data and the those of the filtering outputs. The SMTM, ATTM, and MDTM outputs have RMSE of 0.0415, 0.0412, 0.0332, respectively. The STM output, however, has a much lower RMSE of 0.0227. These results indicate the capability of the proposed STM filter to enhance spatial coherence while preserving considerable resemblance to the input data.

### 3.3. Marine Data

**Figure 8:**
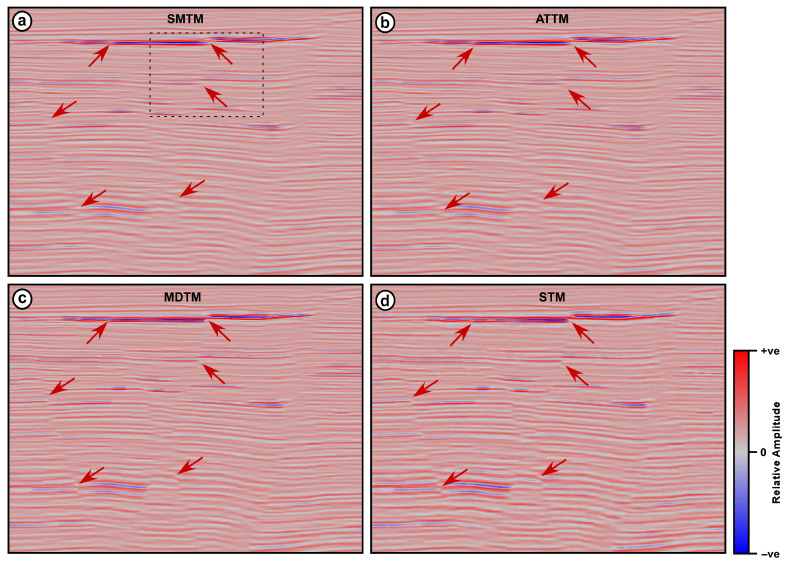
DUG Insight marine seismic section after applying different trace mixing filters (**a**) SMTM, (**b**) MDTM, (**c**) ATTM, and (**d**) STM. The red arrows point to areas of significant differences, and the dotted black rectangle indicates the area magnified in Figure 9.

The second seismic section is a part of marine seismic data collected over a highly faulted area (Figure 3). The section displays a group of steeply dipping, intersecting normal faults that form a graben in the middle of the section. This seismic section is subjected to the above-mentioned four trace mixing filters, and the results are shown in Figure 8. An operator width of 7 traces is used for the four filters, while an alpha value of 0.25 is used for both the ATTM filter and for calculating the reference value in the STM filter.

Both the SMTM and the ATTM filters have resulted in seismic sections in which reflections are smeared and distorted. Moreover, the fault signatures are partially obliterated along the entire section (Figure 8). This phenomenon appears clearly in the shallow strong reflection, which travels across the entire section with a small vertical displacement near its middle. This reflection is so fuzzy on the outputs of the SMTM and ATTM filters that it is hard to distinguish the vertical displacement caused by faulting. The same can be seen in the group of weaker parallel reflections below this strong reflection and along the same fault plane. In general, both the SMTM and ATTM have resulted in a degraded seismic section, where reflections are smeared and interfere with each other as they cross the fault lines.

**Figure 9:**
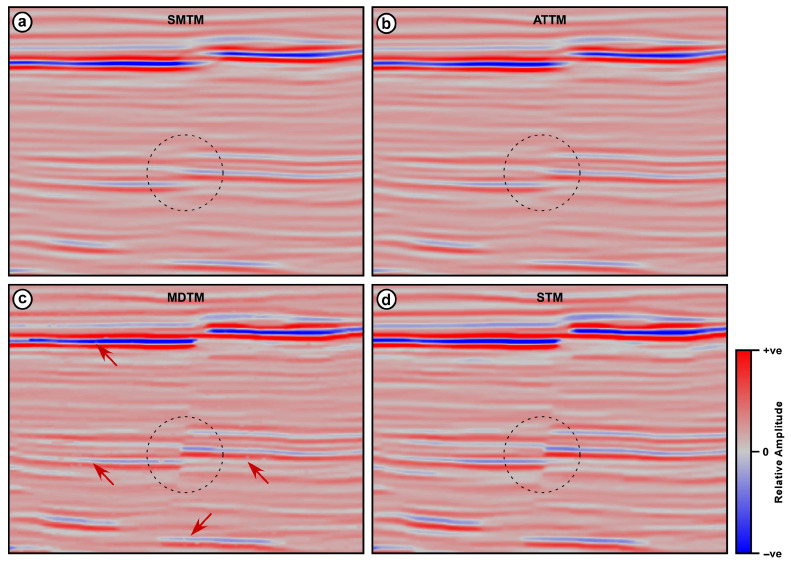
Magnified views of the areas marked with a dotted black rectangle in Figure 8, showing different filtering outputs (**a**) SMTM, (**b**) MDTM, (**c**) ATTM, and (**d**) STM. The dotted black circle indicates the faulted layers, and the red arrows point to areas with rough reflections with inconsistent amplitudes.

On the other hand, the results of the MDTM and STM filters display distinct, well-defined fault planes that clearly offset the seismic reflections. Moreover, faults themselves appear as sharp discontinuities in the reflections. The fault planes can be depicted across the full vertical extent of the section (Figure 8). The red arrows indicate the locations where significant differences between the outputs of the four trace mixing filters can be easily observed. The area marked by the dotted black rectangle in Figure 8 is magnified and displayed in Figure 9. The dotted black circle shows reflection termination near the fault plane. This is a sudden and sharp termination in the STM and MDTM filter outputs, while it is smearing and interfering in the SMTM and ATTM outputs.

The MDTM filter output, however, shows drastic variations in amplitude and waveform along all reflections. These variations are artifacts induced by the filter due to its reliance on a single value, which can result in significant differences among consecutive traces. The red arrows in Figure 9c point to the locations where the waveform and amplitude have a significant difference in the MDTM output, which is not the case in other outputs.

**Figure 10:**
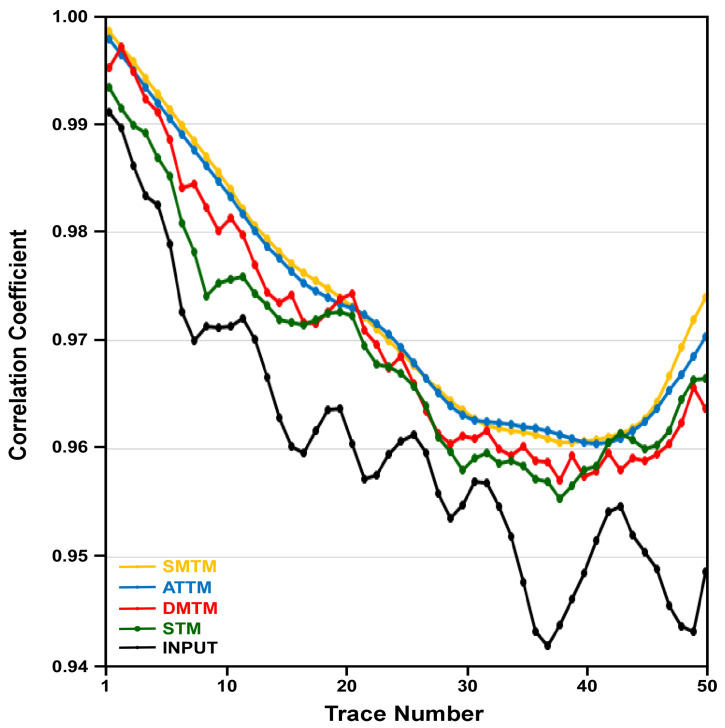
Plot of correlation coefficient between adjacent traces within a selected region with almost horizontal reflections in the input data and the outputs of the four filters for Dug Insight Section.

In general, both the MDTM and the proposed STM filters provide better results than the SMTM and the ATTM filters in terms of reflection coherence and fault clarity. The reflection patterns on either side of the faults show clear vertical displacement, indicating the extent of the fault offset. The sense of motion on the faults can be easily inferred from the relative positioning of the reflectors across the fault planes. However, the MDTM output exhibits some distinct artifacts, which appear as sudden changes in amplitude and waveform along reflections. This type of noise is characteristic of all median filtering tools and is attributed to the fact that the median value can vary significantly from one sample to the next. The STM filter output, however, exhibits the clearest and most enhanced version among the different outputs. The section resulting from STM filtering shows clear termination of reflections on both sides of the fault plane, indicating the precise location of the fault. In the STM output, the lateral coherency of the reflections is enhanced, the spatial variation in amplitude along each reflection is preserved, and the fault signature is improved. As in the previous seismic data, correlation coefficients between adjacent traces are calculated for the input data and the four filtering outputs, as a measure of lateral coherency. Correlation coefficients are calculated for an area with mostly horizontal reflections that lie between the time intervals of 2800 and 3000 ms and CMP numbers 201 and 250. The correlation coefficient is calculated between every two adjacent traces in the input section and the four filtering outputs, and the results are plotted in Figure 10.

Both the SMTM (yellow) and ATTM (blue) filters have over-smoothed the reflections, resulting in correlation coefficients that appear as two identical smoothed curves, which are hardly comparable to the curve of the input data. These two curves exhibit a significant enhancement in the correlation coefficients, but they bear no resemblance to the input curve. This implies aggressive mixing at the expense of the individual trace characteristics. The correlation coefficient curve of the MDTM output, however, is slightly analogous to the input curve, but it contains some kind of irrelevant noise spikes that distort the correlation coefficient curve. These noise spikes are attributed to the fact that the median undergoes drastic changes between neighboring samples, and hence between neighboring traces. This is attributed to the median’s reliance on the single median value. On the contrary, the correlation coefficient curve of the STM output shows a reasonable enhancement in correlation coefficient values compared to the input data.

The correlation coefficient curve of the STM output maintains a high degree of comparability with the original input curve (Figure 10). This can be attributed to the operational mechanism of the STM filter, which enhances lateral coherency while preserving the original relationships between the traces under processing.

Root Mean Square Error (RMSE) is calculated between the input data and the SMTM, ATTM, MDTM, and STM outputs. The SMTM and the ATTM outputs have a relatively high RMSE of 0.103 and 0.096, respectively. The MDTM has an RMSE of 0.078, while the STM has the lowest RMSE of 0.068, proving the ability of the STM filter to preserve relatively high resemblance to the input data.

Incoherence is a crucial seismic attribute and a valuable tool for identifying and characterizing faults and other subsurface structural discontinuities. It is a measure of the lateral variability of the seismic amplitude and waveform across a seismic section, and accordingly, it quantifies the degree to which adjacent seismic traces are different from one another [20,21]. The incoherence attribute is calculated by comparing the seismic waveform on each trace to its neighbors, looking for abrupt changes or disruptions in the reflection patterns. This is typically carried out using either trace-to-trace correlation analysis or gradient-based edge detection. Areas with high incoherence values correspond to zones of structural disruption, such as faults, fractures, channels, mass transport deposits, and other geological discontinuities [22].

The advantage of the incoherence attribute is that it can often detect subtle faults and stratigraphic features that may not be easily visible on the original seismic amplitude data alone. Overall, the incoherence attribute is a powerful analytical tool that can significantly enhance a seismic interpreter’s ability to identify and interpret subsurface structural features from seismic data [23,24,25].

**Figure 11:**
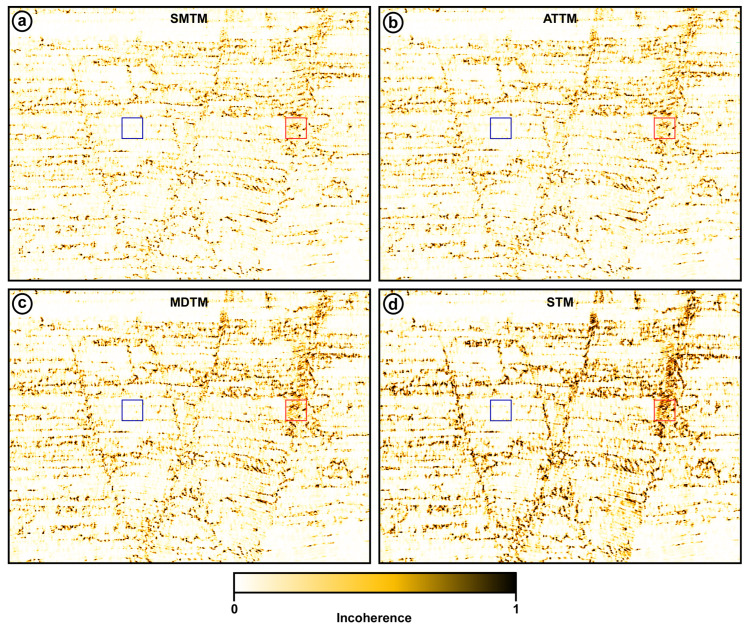
Incoherence seismic attribute of the outputs of the four trace-mixing filters (**a**) SMTM, (**b**) ATTM, (**c**) MDTM, and (**d**) STM. The red and blue squares represent high- and low-incoherence regions used to compare the incoherence ratio among the different outputs.

Figure 11 shows the incoherence attribute for the outputs of the four trace mixing filters. This figure shows that the STM output is significantly better than the remaining three outputs. The incoherence attribute of the STM shows an image in which all faults are delineated and detected. Moreover, a fault plane that can hardly be depicted on the original amplitude section can be easily noticed in the STM incoherence attribute (Figure 11).

Quantitative assessment of incoherence attributes facilitates credible evaluation of the outputs of examined trace mixing tools. The mean incoherence attribute is calculated within two 50 × 50 squares for the four trace mixing outputs. One is the red square placed on a major fault region and the other is the blue square placed in an area with only background. The incoherence attribute ratios are calculated by dividing the mean incoherence of the fault region by the mean incoherence in the background region. The results are listed in Table 2.

The mean background incoherence values show no significant variation among the 4 filtering outputs, with values around 0.04. On the other hand, the mean coherence in the fault region shows remarkable variation, with the STM output yielding the highest value of 0.8832. The other three filters yield close moderate values of about 0.5. Unsurprisingly, the STM output has the highest incoherence ratio of 21.385. The SMTM, ATTM, and MDTM filters have much lower incoherence ratios of 12.438, 12.510, and 14.285, respectively. These results confirm the capability of the STM filter to preserve signal reflected by complex structure compared to other conventional trace mixing tools.

## 4. Discussion

Trace mix filtering is a useful seismic data processing tool that enhances the coherency and lateral continuity of seismic reflections. Despite their ability to improve clarity and, hence, the interpretability of seismic data, these filters are rarely applied to post-stack seismic data for two reasons. The first reason is that seismic data at this stage already have a high enough signal-to-noise ratio, which reduces the benefits of the trace mixing process. The second reason is that the trace mixing process may introduce artifacts, which can damage the reflection discontinuities and obscure genuine structures, such as faults. Trace mixing may also smear reflections, making the auto-detection tools and even manual interpretation of seismic data less successful.

Tests conducted on synthetic data show that the performance of the proposed STM filter stands out significantly when compared to the other three conventional filtering tools, particularly in terms of output clarity and reflection continuity. The STM filter excels in highlighting features, as exemplified by the well-defined dipping reflections, which are largely obscured or distorted in the outputs of MDTM, SMTM, and ATTM filters. This clarity extends to weak dipping reflections, underscoring the STM’s superior ability to maintain reflection integrity. Quantitative evaluations further affirm the STM’s superiority, as indicated by its lowest RMSE value and highest correlation coefficient with noise-free reference data, positioning it as a promising tool for more accurate and detailed data interpretation in filtering processes. Although synthetic data may not fully capture the intricacies of real-world variability, they provide a robust framework for preliminary assessments of filtering performance.

The performance of existing trace mix filters is nearly perfect when applied to seismic data with horizontal layers and no abrupt changes in geology, resulting in no vertical shifts or dipping of reflections. In structurally complicated areas such as faulted layers, however, both the SMTM and the ATTM filters cause smearing of reflections along the crossover point of faults. The reason for this phenomenon is that as the stationary filter operator slides along faulted layers, samples from both sides of the fault are averaged together. This averaging of samples belonging to different reflections results in blurring of the fault trace itself and reduces its interpretability. A good example of this harmful effect can be seen in Figure 6a,b, in areas marked with dotted black circles in Figure 9a,b. The over-smoothing effect of both filters is also evident in the correlation coefficients between adjacent traces, as shown in Figure 7 and Figure 10. Decreasing the operator width of these conventional filters can slightly reduce this harmful effect. However, it reduces the effectiveness of the filter itself, while it does not eliminate the smearing and blurring in the faulted region.

The MDTM filter does not suffer from the previously mentioned smearing and interference issue because no averaging is involved in the filtering process. The filter relies on a single median sample at each stage of the filtering process. As the operator slides over the fault trace, the median changes and indicates a new value representing the new set of samples on the other side of the fault trace. However, this comes at the expense of the overall quality of the processed data. Because the median trace mix relies on only one sample value, this value usually changes drastically even with a single step, as the filter operator slides either vertically or horizontally. This drastic change results in abrupt distortions in reflection amplitudes and rough reflection edges. This type of change can be observed in Figure 6c and in the regions marked by the red arrows in Figure 9c. The MDTM filter introduces waveform distortion along reflection that degrades the overall quality of the output seismic data. The correlation coefficients between adjacent traces, as shown in Figure 7 and Figure 10, also indicate that the largest changes occur between consecutive traces in the output of the MDTM filter.

The proposed STM filter produces a seismic section of better quality than the original seismic section and the output of the three conventional trace mixing filters. In the Nara land seismic section, faults are well-defined, reflection coherency is enhanced, and no features are lost. Moreover, the sag and drag effect of the fault on the ductile sedimentary layers is clear, as the partial folding of these layers is preserved, unlike conventional filters. In the DUG Insight marine seismic section, both faults and reflections are well-distinguished, and no smearing occurs for reflections crossing the fault plane in the STM output, unlike the rest of the trace mixing filters.

The major limitation of the proposed STM filter is its heavy computational load, as new calculations for a reference sample, excluding samples, weighting, and normalization are required at each step of the operator filter. The computational time required for the proposed filter is much longer than that for other stationary trace mix filters, where the same process with the same parameters is applied to the entire seismic section. The computational time required for the SMTM, ATTM, MDTM, and STM for the second dataset are 0.85, 1.34, 0.92, and 8.66, respectively. These computational times are estimated on an Intel Core i7-4770 CPU, 3.40 GHz computer utilizing MATLAB 2023b software. These are significantly large differences considering the processing of an 800 × 1000 matrix of seismic data. However, given the computing power available today and its expected logarithmic increase in the future, the computational load of the proposed filter can be considered trivial.

In conclusion, experiments conducted on the proposed STM filter against the three existing filters (SMTM, MDTM, and ATTM) show that the STM filter yields the best output. The selective STM filter enhances seismic section clarity, improves reflection coherency, and increases the interpretability of seismic data, especially in areas with complex subsurface structures. However, implementing a new seismic filter requires careful cost/benefit analysis. Development and computational costs, including processing time, specialized hardware, training time and cost, and potential workflow disruptions, must be weighed against tangible benefits like enhanced signal clarity, improved structural resolution, and reduced interpretation risks brought by the STM filter. While the STM filter yields superior subsurface images that justify exploration and development decisions, it is critical to decide when to use such a computationally heavy filter. In complex reservoirs, such enhancements are vital and can significantly reduce misinterpretation and drilling uncertainty.

Our future studies will focus on investigating the impact of various filter parameters, such as the operator width, the trimming percentage, and the weighting factor on the performance of the STM filter. By systematically varying these parameters, we aim to determine how they influence noise reduction and signal preservation in both synthetic and real seismic data. Another limitation of the proposed STM filter is its effectiveness when applied to seismic data with steeply dipping reelections. Therefore, our future research will examine the effectiveness of the STM filter on horizons having different dip angles.

## 5. Conclusions

The STM filter proposed in this study is a data-dependent trace mixing filter that addresses the shortcomings of commonly used conventional trace mix filters. STM filter changes spontaneously based on the values and diversity of amplitudes within the operator. This filter analyzes amplitudes within the operator window, automatically excludes samples that may cause harmful effects on the mixing process, and reduces the contribution of trivial and irrelevant amplitudes before performing the mixing process. This addresses the stationarity issue inherent in the currently used trace mixing tools. This problem is the major disadvantage of existing trace mix filters, as they neglect the non-stationary nature of seismic data both laterally and vertically. The filter also guarantees that only relevant data is included in the mixing process. Consequently, selective trace mix provides an output with the advantages of the mixing process while eliminating its downsides in a data-dependent manner. Both the exclusion and weighting processes are changing as the filter operator slides in both directions to accommodate the temporal and spatial variant nature of seismic data. This STM filter can be applied to post-stack seismic data with confidence, unlike the commonly used conventional trace mixing filters, which are rarely applied to post-stack seismic sections because their harm may outweigh their benefits. Future work on the effect of varying STM parameters such as the filter width and exclusion ratio is expected to enhance the performance of the STM filtering. Another future topic of interest is to evaluate the STM filter performance when applied to dipping reflections with variable dip angles.

## Figures and Tables

**Table 1:** Root Mean Square Errors and correlation coefficients between the noise-free synthetic data and the 4 filtering outputs.

	SMTM	ATTM	DMTM	STM
Root Mean Square Error	1.7302	1.6548	1.0389	0.9750
Correlation Coefficient	0.7161	0.7185	0.6927	0.8178

**Table 2:** Mean incoherence within the fault region, a background region, and the incoherence ratio for the four filtering outputs.

	SMTM	ATTM	DMTM	STM
Fault Incoherence	0.5112	0.5229	0.5757	0.8832
Background Incoherence	0.0411	0.0418	0.0403	0.0413
Incoherence Ratio	12.438	12.510	14.285	21.385

## Data Availability

The data presented in this study are not available, while MATLAB codes of the conventional and the proposed STM filters are available upon request and can be acquired by contacting the corresponding author.
